# Diagnostic efficacy of serum cytokines and chemokines in fungal bloodstream infection in febrile patients

**DOI:** 10.1002/jcla.23149

**Published:** 2020-01-23

**Authors:** Qi Wang, Ming Yang, Chi Wang, Jiayue Cui, Xinjun Li, Chengbin Wang

**Affiliations:** ^1^ Department of Clinical Laboratory Medicine Chinese PLA General Hospital & Medical School of Chinese PLA Beijing China; ^2^ Department of Orthopedics Chinese PLA General Hospital & Medical School of Chinese PLA Beijing China; ^3^ Department of Clinical Laboratory The Third Xiangya Hospital of Central South University Changsha China; ^4^ School of Laboratory Medicine and Life Sciences Wenzhou Medical University Wenzhou China

**Keywords:** biomarkers, chemokine, cytokine, fungemia

## Abstract

**Background:**

The role of serum cytokines/chemokines in early diagnosis of fungal infections has not been clearly clarified yet. This study aims to measure the serum levels of cytokines/chemokines in cases of fungemia and to compare them with culture‐negative controls.

**Methods:**

In total, fourteen types of serum cytokines and chemokines from 41 patients with fungemia were compared with 57 patients with negative blood culture results. The cytokine and chemokine levels were detected with multiplex platform. We then performed statistical analysis as a two‐tailed *P* < .05. ROC analysis was performed, and an area under the curve (AUC), and sensitivity and specificity values were calculated to determine the efficacy of various cytokines and chemokines for fungemia. Binary logistic regression was performed to further explore the combination mode of cytokines and chemokines, which could increase the diagnostic efficiency.

**Results:**

C‐reactive protein and procalcitonin were significantly higher compared with those in negative control group, while white blood cell, percentage of neutrophil, percentage of lymphocyte, and ratio of neutrophil and lymphocyte did not differentiate between two groups. Serum levels of IFN‐γ, TNF‐α, MIP‐1β, IL‐6, IL‐8, IL‐10, IL‐12p70, and IL‐17 were significantly higher in patients with fungemia compared with the control group. Combination of MIP‐1β and IL‐17 could improve the AUC, sensitivity, and specificity for the diagnosis of fungemia.

**Conclusion:**

Our study demonstrates that serum cytokines and chemokines including IFN‐γ, TNF‐α, MIP‐1β, IL‐6, IL‐8, IL‐10, IL‐12p70, and IL‐17 could be considered as diagnostic markers for fungemia. Combination of these biomarkers might improve the diagnostic efficiency of fungemia.

## 1 INTRODUCTION

Increasing incidence of fungemia among nosocomial bloodstream infections has been observed over the recent years due to increase of immunocompromised individuals with systemic diseases such as diabetes mellitus and acquired immune deficiency syndrome.^1^, ^2^ Fungemia is a life‐threatening condition with the global mortality rate surging more than fivefold in the past decade.^3^, ^4^ It has been reported that the outcome depends on early and targeted treatment; thus, prompt diagnosis is essential at the early stage of infection.

Currently, early diagnosis of fungemia remains a complicated issue.^5^ Although blood culture remains the reference method for the detection of fungemia, this method takes 3‐5 days to generate positive results and also suffers from low sensitivity, which could not meet the requirement of early diagnosis and clinical guidance for fungemia. In addition, matrix‐assisted laser desorption/ionization time‐of‐flight mass spectrometry (MALDI‐TOF‐MS) has been used for the diagnosis of pathogens in bloodstream infections, with a shorter turnaround time.^6^ However, MALDI‐TOF‐MS also depends on the colony originated from blood culture, thus limiting its application. Molecular methods such as PCR assays and next‐generation sequencing (NGS) have also been developed during the past years. But due to multiple manual operations in the whole process, the lack of conclusive validation for commercially available assays, and variety of methodologies, these methods have not been recommended for routine use in clinical practice.^7^ Serological marker detection allows for a short turnaround time and hence is an important tool for early diagnosis of fungemia.

Cytokine analysis has been performed in bacteremia,^8^ and cytokine release varies by the pattern. Studies have shown that the concentrations of cytokines/chemokines may increase earlier than currently available inflammatory proteins such as C‐reactive protein (CRP) and procalcitonin (PCT). Thus, these cytokines/chemokines may be considered as diagnostic biomarkers for fungal bloodstream infection.^9^

Thus, our study aims to measure the serum levels of cytokines including interleukin (IL) ‐1β, ‐2, ‐3, ‐4, ‐6, ‐8, ‐10, ‐17, ‐12p70, granulocyte colony‐stimulating factor (G‐CSF), interferon (INF)‐γ, and tumor necrosis factor (TNF)‐α; chemokines including macrophage inflammatory protein‐1β (MIP‐1β) and regulated on activation normal T cell expressed and secreted (RANTES)/chemokine (C‐C motif) ligand 5 (CCL5). In addition, currently available clinical laboratory parameters including white blood cell (WBC), percentage of neutrophil (N%), percentage of lymphocyte (L%), neutrophil‐to‐lymphocyte ratio (NLR), C‐reactive protein (CRP), and procalcitonin (PCT) were also collected from the database in cases of fungemia and to compare them with those of culture‐negative individuals.

## 2 MATERIALS AND METHODS

### 2.1 Patient selection and group division

Patients with blood cultures that yielded fungi and patients with negative blood culture results were included in our study. All these enrolled patients were suspected of infection due to their increased temperature from fever clinics or different departments of Chinese PLA General Hospital between January 2016 and December 2018. Informed consent was obtained from all patients. Since the number of patients with fungal bloodstream infections was not very large, these patients were consecutive. However, for the negative results patients, since the negative rate is quite high, these patients were not consecutive and we chose patients with matched age and sex with culture‐positive patients. This study was reviewed and approved by the Ethics Committee of Chinese PLA General Hospital (S2018‐207‐02). Patients with positive blood culture results were considered as the fungemia group and patients with negative culture results as the control group.

### 2.2 Blood culture and identification of pathogens

Blood samples (8‐10 mL) for culture were taken from the patients for two to three times and inoculated using BACTEC PLUS Aerobic/F (BD) blood culture bottles. The isolations from blood culture were identified to species with VETIK YBC card (Bio‐Merieux) and MALDI‐TOF‐MS (Bio‐Merieux).

### 2.3 Sample collection

All patients underwent blood collection during the early stage of onset (within 48 hours of admission) and before the use of antibiotics. Serum samples were obtained at the simultaneous time of blood culture and then separated immediately with the centrifuge (Thermo‐Electron Corporation) at 1500 *g* for 5 minutes. Then, the supernatant was transferred to polypropylene tubes (Solarbio) and stored at −80° refrigerator before analysis.

### 2.4 Measurement of the serum cytokine levels

The serum samples were analyzed using a Luminex^®^ xMAP Bead Array Platform (Millipore Corporation). A combination of 14 cytokines and chemokines was quantified using the reagents of selected ones from Human Cytokine 30‐Plex Panel Kit. Evaluated cytokines included the following: IL‐1β, ‐2, ‐3, ‐4, ‐6, ‐8, 10, 17, 12p70, INF‐γ, TNF‐α, G‐CSF, RANTES, and MIP‐1β.^10^ The assays were conducted according to the instruction of instrument, and data were analyzed with Milliplex analyte v5.1.0.0 software.

### 2.5 Measurement of serum CRP and PCT

CRP was measured using nephelometric method (Siemens BNII, Cardiophase), and PCT was measured by Cobas800 (Roche) based on electrochemical luminescence technology.

### 2.6 Statistical analysis

Data were analyzed with SPSS 22.0 and GraphPad Prism 5 (GraphPad Software). The distribution of each continuous variable was compared with the normal distribution using the Shapiro‐Wilk test. For non‐normally distributed data, the median and quartiles were used to represent concentrated and discrete trends, and Mann‐Whitney *U* test was used as a nonparametric test to compare samples between two groups. In order to determine the cutoff value for cytokine serum levels, receiver operating characteristic (ROC) analysis was performed, and an area under the curve (AUC), and sensitivity and specificity values were calculated. Statistical significance was assumed based on a value of *P* < .05.

## 3 RESULTS

### 3.1 General characteristics of enrolled patients

From 2016 to 2018, 41 patients had an episode of microbiologically proven fungal infection. Another 54 culture‐negative patients were enrolled as the control group. The average age, ratio of male/female, and the distribution of primary diagnoses are illustrated in Table 1. Among the pathogens detected by blood culture, *Candida albicans* had the highest positivity rate of 14/41 (34.1%), followed by *Candida glabrata* 11/41 (26.8%), *Candida tropicalis* 8/41 (19.5%), *Candida parapsilosis* 7/41 (17.1%), and *Trichosporonsis* 1/41 (2.4%) (Table 2).

**Table 1:** Demographical and clinical characteristics of enrolled patients

	Fungemia (n = 41)	Non‐fungemia (n = 54)	*P* value
Age (y)	58.63 ± 21.97	63.78 ± 19.11	NS
Male/Female	16/25*	33/21	<.05
Primary diagnosis [n (%)]			
Leukemia or non‐Hodgkin's lymphoma	2 (4.9%)	1 (1.8%)	NS
Pancreatitis or pancreatic lesions	8 (19.5%)*	2 (3.5%)	<.05
Urinary system infections	4 (9.6%)*	2 (3.7%)	<.05
Cardiovascular system disease	7 (17.1%)*	3 (5.6%)	<.05
Obstructive jaundice or Biliary tract infection	3 (7.3%)	3 (5.6%)	NS
Hepatic disease	2 (4.9%)	1 (1.8%)	NS
Intestinal obstruction	2 (4.9%)*	0	<.05
Abdominal pain or related symptoms	2 (4.9%)	2 (3.7%)	NS
Cancer	5 (12.2%)	2 (3.7%)	NS
Nervous system disease	2 (4.9%)	1 (1.9%)	NS
Musculoskeletal system disease	2 (4.9%)	1 (1.9%)	NS
Respiratory system disease	1 (2.4%)**	24 (44.4%)	<.01
Immune system disease	0	2 (3.7%)	NS
Impetigo	0	1 (1.9%)	NS
Infection of parapharyngeal space	0	1 (1.9%)	NS
Trauma	0	1 (1.9%)	NS
Fever or sepsis or shock	1 (2.4%)**	10 (17.5%)	<.01

* Indicates a significant statistical difference compared with the non‐fungemia group (*P* < .05).

** Indicates a significant statistical difference compared with the non‐fungemia group (*P* < .01).

**Table 2:** Microbiological distribution of the fungemia groups

Causative pathogens	Patients, n (%)
Total	41
Candida albicans	14 (34.1%)
Candida glabrata	11 (26.8%)
Candida tropicalis	8 (19.5%)
Candida parapsilosis	7 (17.1%)
Trichosporonsis	1 (2.4%)

### 3.2 Comparison of commonly used diagnostic parameters between two groups

Commonly used diagnostic biomarkers in clinical laboratory including white blood cell (WBC), percentage of neutrophil (N%), percentage of lymphocyte (L%), and ratio of neutrophil and lymphocyte (NLR) did not differentiate between the fungemia group and the negative control group (Table 3). In the group with fungal infections, mean level of CRP was (11.31 ± 6.86) mg/dL; median level of PCT was 1.15 (interquartile range: 0.41‐10.22) ng/mL, while in the control group, mean level of CRP was (7.90 ± 6.44) mg/dL; median level of PCT was 0.34 (interquartile range: 0.12‐2.98) ng/mL. Both CRP and PCT were significantly higher in the fungemia group when compared with those in the control group (Table 3). Sensitivity of CRP was 66.7%, and specificity was 71.1% at the cutoff value of 8.575 mg/dL and an AUC of 0.655 (95% CI: 0.556‐0.817). Sensitivity of PCT was higher (88.9%), while specificity was 47.2% at the cutoff value of 0.3035 pg/mL and an AUC of 0.699 (95% CI: 0.571‐0.827).

**Table 3:** Comparison of laboratory parameters between fungemia and culture‐negative groups

	WBC (10^9^/L)	N	L	NLR	CRP (mg/dL)	PCT (ng/mL)
Fungemia	10.46 ± 7.37	0.84 ± 0.10	0.10 ± 0.08	8.86 ± 14.79#	11.31 ± 6.86*	1.04 ± 9.81*, #
Non‐fungemia	9.28 ± 7.11	0.80 ± 0.11	0.13 ± 0.08	9.15 ± 10.19#	7.90 ± 6.44	0.34 ± 2.86*, #

Abbreviations: CRP, C‐reactive protein; L, percentage of lymphocyte; N, percentage of neutrophil; NLR, ratio of neutrophil/lymphocyte; PCT, procalcitonin; WBC, white blood cell.

* Indicates a significant statistical difference compared with culture‐negative group (*P* < .05).

^#^ Data were presented as median ± interquartile range.

### 3.3 Comparison of fourteen cytokines and chemokines between two groups

Fourteen cytokines and chemokines were analyzed at the early time points prior to initiation of antifungal therapy. Serum concentrations of IFN‐γ, TNF‐α, MIP‐1β, IL‐6, IL‐8, IL‐10, IL‐12p70, and IL‐17 were significantly higher in patients with the fungemia group compared with the control group, while the other six cytokines/chemokines did not differ between two groups (Table 4).

**Table 4:** Comparison of serum cytokine levels between the fungemia group and the negative control group

	Fungemia	Culture‐negative	*P*‐ value
IFN‐γ, pg/mL, median (interq. interval)	25.53 (8.40‐221.57)	6.47 (2.945‐21.91)	.000***
G‐CSF, pg/mL, median (interq. interval)	70.55 (40.6‐218.96)	52.19 (30.57‐114.12)	.168
TNF‐α, pg/mL, median (interq. interval)	54.18 (34.67‐96.14)	23.33 (14.7‐39.34)	.000***
MIP‐1β, pg/mL, median (interq. interval)	90.72 (59.35‐150.49)	50.56 (35.78‐65.63)	.000***
RANTES, pg/mL, median (interq. interval)	21332.0 (7185‐58889)	19825.5 (6328.6‐45099)	.674
IL‐1β, pg/mL, median (interq. interval)	2.35 (1.55‐4.93)	3.15 (1.44‐5.41)	.400
IL‐2, pg/mL, median (interq. interval)	2.38 (1.41‐5.15)	2.18 (1.27‐3.03)	.301
IL‐3, pg/mL, median (interq. interval)	0.93 (0.49‐1.61)	1.1 (0.55‐2.10)	.626
IL‐4, pg/mL, median (interq. interval)	18.6 (10.96‐34.46)	14.43 (9.17‐25.49)	.191
IL‐6, pg/mL, median (interq. interval)	85.52 (25.64‐247.46)	41.11 (12.17‐25.48)	.045*
IL‐8, pg/mL, median (interq. interval)	67.09 (38.34‐147.22)	38.58 (12.17‐71.67)	.002**
IL‐10, pg/mL, median (interq. interval)	144.47 (53.68‐456.48)	17.05 (5.64‐49.93)	.000***
IL12p70, pg/mL, median (interq. interval)	6.60 (2.53‐13.71)	3.09 (1.11‐4.54)	.004**
IL17, pg/mL, median (interq. interval)	6.38 (3.62‐16.96)	2.90 (1.84‐4.54)	.000***

* *P* < .05.

** *P* < .01.

*** *P* < .001.

ROC curve analysis indicated that IL‐10 had the highest AUC (area under the curve) for differentiating patients with fungemia from culture‐negative patients: 0.792 (95% CI: 0.651‐0.889). The AUC for distinguishing between fungemia and culture‐negative patients using IFN‐γ, TNF‐α, IL‐8, IL‐12p70, and IL‐17 was 0.719 (95% CI: 0.589‐0.849), 0.753 (95% CI: 0.631‐0.874), 0.671 (95% CI: 0.544‐0.816), 0.717 (95% CI: 0.589‐0.845), and 0.773 (95% CI: 0.646‐0.879), respectively. Sensitivity of IL‐6 was the highest (96%), while its specificity was only 25% with the cutoff value of 12.11 pg/mL and an AUC of 0.574 (95% CI: 0.427‐0.712). Specificity of MIP‐1β was the highest (79.4%), and its sensitivity was 77.8% at the cutoff value of 61.82 pg/mL and an AUC of 0.738 (95% CI: 0.613‐0.863). AUC, sensitivity, specificity, and cutoff values of all significantly increased cytokines and chemokines for predicting fungemia patients are illustrated in Table 5.

**Table 5:** Diagnostic parameters of cytokines and chemokines for fungemia

	Sensitivity (%)	Specificity (%)	Cutoff (pg/mL)	AUROC (95% CI)
IFN‐γ	66.7	72.2	22.195	0.719 (0.589‐0.849)
TNF‐α	81.5	64.1	34.455	0.753 (0.631‐0.874)
MIP‐1β	77.8	79.4	61.82	0.738 (0.613‐0.863)
IL‐6	96.3	25	12.11	0.574 (0.427‐0.712)
IL‐8	66.7	66.7	56.275	0.671 (0.544‐0.816)
IL‐10	77.8	75	51.525	0.792 (0.651‐0.889)
IL12p70	66.7	69.4	6.13	0.717 (0.589‐0.845)
IL‐17	81.5	63.9	3.445	0.773 (0.646‐0.879)
CRP	66.7	71.1	8.575*	0.655 (0.556‐0.817)
PCT	88.9	47.2	0.3035#	0.699 (0.571‐0.827)
MIP‐1β + IL‐17	81	81.5	‐	0.863 (0.787‐0.939)

Abbreviations: AUROC, area under the receiver operating characteristic.

* Unit for CRP is mg/dL.

^#^ Unit for PCT is ng/mL.

With binary logistic regression, MIP‐1β and IL‐17 comprised the best performing model. The logistic model parameter estimates are found in Table 6. The AUC, sensitivity, and specificity of combination of MIP‐1β and IL‐17 were 0.863 (95% CI: 0.787‐0.939), 81%, and 81.5%, respectively (Table 5).

**Table 6:** Logistic model parameter estimates and odds ratios

Cytokine	Estimates	SE	Wald	*P*	OR	95% CI
IFN‐γ	0.009	0.005	3.671	.055	1.009	1.000‐1.018
MIP‐1β	0.018	0.009	4.137	.042*	1.018	1.001‐1.036
IL‐17	0.127	0.056	5.100	.024*	1.136	1.017‐1.269
TNF‐α	−0.002	0.005	0.213	.644	0.998	0.987‐1.008
IL‐10	−0.001	0.001	0.963	.326	0.999	0.998‐1.001
IL‐12p70	−0.027	0.019	1.971	.160	0.973	0.937‐1.011

Abbreviations: CI, confidence interval; OR, odds ratio; SE, standard error. * p<0.05

## 4 DISCUSSION

With the extensive use of broad‐spectrum antibiotics, the increase of immunosuppressive population, and development of invasive treatment technology, the incidence of fungal bloodstream infection rises gradually in the past decade.^10^ Currently, successful treatment of fungal infection requires cooperation of entire healthcare team; thus, systemic therapy should be applied once the diagnosis is defined. Innate immune cells including neutrophils and macrophages are the first line of host defense, which produce a number of pro‐inflammatory cytokines and chemokines to eliminate pathogens upon their entry.^11^ Netea et al^12^ reported that *C albicans* induces cytokine production by binding to different macrophage receptors, indicating that collaborative recognition of distinct fungal components by different classes of innate immune receptors is critical for inflammatory response development. Though several studies have documented changes of cytokines and chemokines in bacteremia or sepsis, few studies were reported in fungemia. Therefore, in our study, we investigated the profiles of various cytokines and chemokines that are involved in the regulation of systemic inflammation and found that serum IFN‐γ, TNF‐α, MIP‐1β, IL‐6, IL‐8, IL‐10, IL‐12p70, and IL‐17 might be useful for early diagnosis of fungemia.

In our study, CRP and PCT significantly increased in the fungemia group compared with negative control, while other commonly used laboratory parameters such as WBC, N%, L%, and NLR did not differ between two groups. As reported by previous studies, serum CRP and PCT levels may be useful for differential diagnosis of sepsis and CRP levels were lower in patients with fungemia than those with bacteremia.^13^, ^14^ In another study, “low PCT and high CRP” were found in case of fungal infections.^15^, ^16^

In the current study, IL‐17 found to be significantly increased in the fungemia group, and after combination with MIP‐1β, these two cytokine/chemokines could improve the diagnostic efficiency (with the sensitivity of 81% and specificity of 81.5%). IL‐17 is produced by Th17 cells, a subset of CD4 + T helper cells, which could induce the expression of other chemokines, cytokines, and matrix metalloproteinases through NF‐κB and MAPK signaling pathways, the latter of which could then propagate cascade events leading to neutrophil recruitment, inflammation, release of antifungal defensins for host defense.^17^ It has been reported that the level of IL‐17 is important for host defense against *Candida* species.^18^ The protective role of Th17 responses in the antifungal host defense was first established in IL‐17 receptor‐deficient mice that showed increased susceptibility to a disseminated *Candida albicans* infection.^19^ In addition, the importance of IL‐17 for the host defense against *Candida* infections has been underlined by the increased number of infectious complications seen in patients with psoriasis who have been treated with IL‐17A‐targeted antibodies.^20^ The level of cytokine IL‐17 has been reported to elevate in the serum samples of patients with candidemia when compared with patients with bacterial sepsis or healthy control subjects.^21^

MIP‐1β is also significantly increased in our study and could be combined together with IL‐17 to improve the diagnostic efficiency. Chemokines play an important role in the trafficking of immune cells during infection.^22^ The C‐C chemokines include molecules include human monocyte chemotactic protein 1 (MCP‐1), RANTES/CCL3, and macrophage inflammatory protein 1α and 1β (MIP‐1α/CCL4 and MIP‐1β/CCL5), which exhibit chemoattactant potential for monocytes but not neutrophils.^23^ Through binding to its receptor (CCR5), MIP‐1β regulates the balance between Treg and Th17 cells and thus weakening pro‐inflammatory response, which could facilitate pathogen clearance during *H capsulatum* infection.^24^ In addition, functions and recruitment of NK cells are mediated by chemokines including MIP‐1α and MIP‐1β during fungal infections.^25^, ^26^ For example, during *A fumigatus* infections, MIP‐1α and MIP‐1β are prominently up‐regulated in NK cells.^27^ In particular, MIP‐1 is regarded as a critical mediator of host defense against *A fumigatus* infection and its single nucleotide polymorphisms are significantly correlated with fungal infections.^28^ Furthermore, chemokines have been detected to be released earlier than cytokines (TNF‐α and IFN‐γ), which indicates that recruitment of macrophages and neutrophils by NK cells is in advance of adaptive immunity by cytokines when counteracting a fungal infection.^27^

IL‐10 is the cytokine with the highest AUC (0.792, 95% CI: 0.651‐0.889) in our study. IL‐10 is released from macrophages and DCs, and the main capacity of this anti‐inflammatory cytokine is to block production of other cytokines from T helper (Th)1 cells.^29^ The balance between IL‐10 and IL‐12 p70 released from DC is critical for immune response, for example, prominent production of IL‐10 accompanied by low production of IL‐12 p70 represents response to fungal patterns.^30^ Armstrong et al reported that immunocompromised individuals could not response to invasive fungal disease through IL‐10 release,^31^ which indicated that high concentrations of IL‐10 represent an effective immune response. In addition, Mario et al reported the different transcriptional programs involved in IL‐10 production by bacterial and fungal pathogen‐associated molecular patterns,^32^ which indicated that there might be different levels of IL‐10 in bacteremia and fungemia.

Interferon‐gamma (IFN‐γ), which is a vital component of the host immune response against intracellular pathogens and is produced by nature killer (NK) cells and T lymphocytes, can promote classical activation of macrophages and result in increased phagocytosis and production of reactive oxygen species.^33^ The production of IFN‐γ by Th1 cells has been shown to be crucial in the control of systemic candidiasis in a murine model.^34^ Through the production of IFN‐γ, NK cells could regulate their antifungal function such as directly killing the organism.^35^ In addition, IFN‐γ could also participate in the functions of adaptive immunity with representative T‐cell responses for the host's defense. It has been reported that IFN‐γ knockout mice and people with impaired IFN‐γ signaling are at significant risk of severe infection with *Candida albican*.^36^ IFN‐γ has also been used to augment the host immune response in cases of HIV‐negative patients with invasive candidiasis infection.^37^

IL‐12 is made up of 2 subunits, IL‐12p35 and IL‐12p40, which together form active IL‐12p70.^38^ IL12p70 links innate immunity with the development of adaptive immunity, which favors Th1 cell differentiation.^25^ IL‐12 is induced by microbial products in monocytes, macrophages, and dendritic cells (DCs) and acts on NK and T cells to induce IFN‐γ.^39^ The balance between Th1 and Th17 populations could control IL‐12 production through Toll‐like receptor (TLR) and dectin‐1.^40^ It has been reported that IL‐12 blockade could reverse the protective response during systemic *C albicans* infection and when IL‐12 was administered during infection, mice demonstrated increased survival and decreased fungal burden in kidneys.^41^ However, administration of IL‐12 during systemic infection increases disease severity due to IFN‐γ‐mediated pathology, which indicates that the combination of IL‐12 and IFN‐γ needs to be finely tuned for optimal fungal clearance.^42^ Furthermore, human DCs infected with *A fumigatus* induced IL‐12p70 production and promoted IFN‐γ production from CD4 + T cells.^43^

In our study, we also measured the serum levels of TNF‐α, IL‐6, and IL‐8 all of which are important pro‐inflammatory cytokines and are also essential factors in innate immunity. IL‐6 is secreted into the bloodstream and induces both B‐cell and T‐cell differentiation as well as production of antibodies and acute‐phase proteins during acute phase of infections.^44^ Circulating IL‐6 could trigger acute‐phase responses to local or systemic acute inflammation; therefore, it has a pivotal role for development on proper innate immune response.^45^ Romani et al reported that IL‐6‐deficient mice are more susceptible to disseminated candidiasis than wild‐type mice, which suggests that IL‐6 release is fundamental during the fungal infection.^46^ In addition, Kovács R et al reported that serum IL‐6 had strong relationship with systemic *C albicans* infection.^47^ IL‐8 is produced by phagocytes and mesenchymal cells exposed to inflammatory stimuli and considered as neutrophil‐activating cytokine.^48^ In vivo, IL‐8 elicits a massive neutrophil accumulation at the site of injection during inflammation and host defense. Zymosan induced the release of IL‐6 and IL‐8, which is stimulated through activating the MAPK and NF‐κB pathways.^49^ Most of the publicized papers reported IL‐8 as an efficient predictor for bacteremia, while quite few papers are concerned with the changes of IL‐8 during fungal infection. In our study, we found an increase of IL‐8 in the fungemia group compared with the control group. Thus, more work is still required for further exploration of IL‐8 in terms of fungal infection.

Tumor necrosis factor‐alpha (TNF‐α) is a fundamental cytokine in mounting an immune response, whereas its high levels are detrimental in inflammatory diseases.^50^ TNF‐α is stimulated to release after activation of phagocytosis, which plays an important role in determining the development of subsequent antifungal adaptive immune responses such as polarization of naïve Th cells into effector Th17 cells.^51^, ^52^ Studies have shown that *A fumigatus* could induce acute inflammation regulated by neutrophils with pro‐inflammatory cytokines (TNF‐α, GM‐CSF, and IL‐1β) and chemokines (MIP‐1).^53^ It has also been reported that TNF‐α enhances host responses to *A fumigatus*, and inhibition on its function might increase susceptibility to *aspergillosis*.^54^ In addition, anti‐TNFα agents have been shown to be associated with increased infection risks for invasive fungal infections, particularly when given late in the overall course of treatment in pediatric patients.^55^ The neutralization of TNF activity could lead to suppression of the production of IFN, promotion of monocyte apoptosis, and prevention of maintenance of granuloma, allowing fungus growth in several organs.^56^

Previous studies have reported that IL‐1β, IL‐2, IL‐3, IL‐4, G‐CSF, and RANTES play an important role in the regulating both immune activation and homeostasis. When infection occurs, these cytokines/chemokines can lead a systemic inflammatory response. However, in this study, the levels of these cytokines/chemokines were not significantly different between two groups (*P* > .05).

Limitations of this study include its retrospective design and the relatively small number of available subjects. This may further limited the ability to find statistical significance among those cytokine options. In addition, the continuous variations of these cytokines were not recorded due to the absence of sample collection. Thus, we could not evaluate the correlation of cytokine changes over time.

## 5 CONCLUSION

In this study, we found eight serum cytokines/chemokines that significantly increased in the fungemia group when compared with the culture‐negative control group. Among them, MIP‐1β and IL‐17 might be given more attention during the diagnosis of fungemia. The utility of these biomarkers to diagnose fungemia dynamically requires to be assessed in further studies.

## CONFLICT OF INTEREST

The authors declare that they have no conflict of interest.
